# Oxidative Stress, Lipid Peroxidation, and Loss of Hyaluronic Acid in the Human Vitreous Affected by Synchysis Scintillans

**DOI:** 10.1155/2019/7231015

**Published:** 2019-10-30

**Authors:** Loredana Bergandi, Oleksii A Skorokhod, Rosalba La Grotta, Evelin Schwarzer, Raffaele Nuzzi

**Affiliations:** ^1^Department of Oncology, University of Torino, Torino, Italy; ^2^Department of Life Sciences and Systems Biology, University of Torino, Torino, Italy; ^3^Eye Clinic Section, Department of Surgical Sciences, University of Torino, Torino, Italy

## Abstract

The aim of this study was to assess the oxidative stress status in eyes affected by synchysis scintillans and to compare it to vitreoretinal disorders without synchysis scintillans. Human aqueous and vitreous humors were obtained during vitrectomy from thirty-seven otherwise healthy patients that were randomly chosen among patients that had to undergo a 25-gauge *pars plana* vitrectomy from the central vitreous cavity, for either synchysis scintillans (*n* = 16) or vitreoretinal disorders without synchysis scintillans (*n* = 21), such as idiopathic epimacular membrane (*n* = 12), macular hole (*n* = 5), or rhegmatogenous retinal detachment (*n* = 4). The redox parameters thiobarbituric acid reactive substances (TBARS), a measurement of lipid peroxidation, nitrite concentration, an estimate of nitric oxide (NO) production, 4-hydroxynonenal (4-HNE)-protein conjugates, a structural protein modification by lipid peroxidation product 4-HNE, and the antioxidative activities of Cu,Zn-superoxide dismutase (SOD), and catalase were measured in aqueous and vitreous humors and compared between synchysis scintillans affected and not-affected patients. TBARS and nitrite levels of the vitreous humor were significantly higher in patients with synchysis scintillans as compared to patients affected by vitreoretinal disorders without synchysis scintillans. Synchysis scintillans patients had significantly lower activities of SOD and catalase both in aqueous and vitreous humors than patients with vitreoretinal disorders without synchysis. The consequently higher lipoperoxide-dependent 4-HNE production in synchysis scintillans was detectable in aqueous and vitreous humors as a significant increased accumulation of 4-HNE-protein conjugates vs nonsynchysis vitreoretinal disorders. Additionally, hyaluronic acid (HA) was significantly decreased in the vitreous body of synchysis scintillans patients. The data consistently show that synchisis scintillans is accompanied by a redox imbalance with increased oxidative modifications of 4-HNE proteins and loss of HA, both of likely importance for remote damages of the retina. It remains to be proven whether a therapeutic strategy which targets oxidative stress may be effective in the treatment of synchysis patients.

## 1. Introduction

Normal aging eye is accompanied by a number of physiological changes in the vitreous gel, that is, a compact, homogeneous, and clear body at birth and that can undergo progressive degeneration characterized by vitreous liquefaction (synchysis) and weakening of the vitreoretinal adhesion between the posterior vitreous hyaloid and the inner limiting membrane. This degeneration, corresponding with a simultaneous decrease in gel volume, a lateral aggregation of the collagen fibrils (syneresis), and a gradual destruction of the collagen-hyaluronate network, may result in posterior vitreous detachment (PVD) [1, 2]. Thus, PVD could increase the risks of major vitreous retinal diseases such as macular holes, epimacular membranes, vitreoretinal traction syndrome, and retinal detachment [3].

Next to vitreous retinal diseases, synchysis scintillans is a rare bilateral condition characterized by the presence of tiny yellowish-white floating crystals of cholesterol in the vitreous. It tends to occur in eyes that have had uveitis or vitreous haemorrhage, and it is a degenerative change. Moreover, also the senile synchysis scintillans, resulting in liquefied vitreous humor and the accumulation of cholesterol crystals within the vitreous, seems to be involved in the onset of PVD due to the possible vitreous instability, even if only a dated study was reported and performed in eyes of autopsied subjects [3].

An imbalance between free radicals production and antioxidant defenses may produce oxidative stress (OS). The most important oxidants are reactive oxygen species (ROS) and reactive nitrogen species (RNS) that have been implicated as causative factors of many diseases and also involved in aging because of their potential to cause oxidative deterioration of DNA, proteins, and lipids [4], resulting in functional impairment, damage, and inflammation. In many vitreoretinal disorders, such as proliferative diabetic retinopathy (PDR), retinitis pigmentosa, age-related macular degeneration, and rhegmatogenous retinal detachment (RRD), OS has been implicated as a factor inducing the development of retinal cellular damage [5].

The aim of the present study was to assess oxidative stress in aqueous and vitreous humors in patients undergoing the 25-gauge *pars plana* vitrectomy for typical vitreoretinal diseases such as macular hole, idiopathic epimacular membrane, or retinal detachment and in patients affected by synchysis scintillans as vitreous disease. Indeed, as these diseases may be sight-threatening conditions, there is growing interest in unveiling their pathogenic mechanisms to potentially lead to novel therapeutic strategies for their prevention.

## 2. Methods

### 2.1. Patients

The study was carried out in accordance with the Declaration of Helsinki for medical research involving human subjects in the period from February 2016 to June 2018 and was authorized by the local Ethical Committee (ref. number: 0018536). Signed and written informed consent was obtained from all patients accepting to be included in this study. All patients went through a baseline ophthalmological examination before laser capsulotomy including measurement of best-corrected visual acuity (BCVA), Goldmann applanation tonometry, and fundus examination.

Thirty-seven otherwise healthy patients were randomly chosen among patients that had to undergo a 25-gauge *pars plana* vitrectomy from the central vitreous cavity, for idiopathic epimacular membrane (IEM) (*n* = 12), macular hole (MH) (*n* = 5), rhegmatogenous retinal detachment (RRD) (*n* = 4), and synchysis scintillans (SS) (*n* = 16). The mean age of the patients affected by vitreoretinal disorders and synchysis scintillans was 74.0 ± 2.8 and 75.9 ± 2.1 years, respectively. Patients did not receive pre- or postoperative medications. The following clinical characteristics were recorded, and patients presenting with one or more of the criteria were excluded from the study: vitreous hemorrhages or hemovitreous, proliferative diabetic retinopathy, taking antioxidant integrators, impossibility of classifying vitreous degeneration or any of the other relevant parameters, infections, malignant neoplasias, renal or hepatic failure, and pathologic levels of blood glucose and fibrinogen.

### 2.2. Reagents

Unless otherwise specified, reagents were purchased from Sigma-Aldrich (Milan, Italy), whereas plasticware was from Falcon (Becton Dickinson, Franklin Lakes, NJ).

### 2.3. Vitrectomy and Preparation of Aqueous and Vitreous Samples

From each patient, an aqueous (130–150 *μ*l) and a vitreous (900–920 *μ*l) sample were collected at the beginning of the 25-gauge *pars plana* minimally invasive vitrectomy with noncontact technique (BIOM) from the central vitreous cavity [6, 7]. Undiluted samples were immediately placed in cryotubes and stored at −80°C until assayed 2 weeks after sampling at the latest. After sample collection, any type of contamination or exposure to light sources can be excluded. The aqueous and vitreous samples from each patient were thawed and centrifuged to eliminate cellular components and debris [8].

### 2.4. Measurement of Thiobarbituric Acid Reactive Substance (TBARS) Production

Following the indication by Yano, the TBARS assay was used to detect the presence of lipid peroxidation products in vitreous samples, only, due to material restrictions of the aqueous sample [9]. As previously reported [8], 600 *μ*l of the thawed clear vitreous supernatant was sonicated with a 10 s burst using a Bandelin Sonopuls HD 2070 sonicator with a titanium microtip MS73 at 30% of maximal potency (corresponding to 60 W), centrifuged at 16,124 ×*g* for 5 min, and 500 *μ*l of supernatant was added to the TBA solution (thiobarbituric acid 0.375% and trichloroacetic acid 30% in 0.5 N HCl). TBARS generation by sonication was excluded. The samples were boiled for 20 min, then rapidly cooled in ice, and centrifuged for 5 min at 14,883 ×*g*. Absorbance on 300 *μ*l of the sample was measured at 532 nm with a Packard EL340 microplate reader (BioTek Instruments, Winooski, VT). Results are expressed as nmol TBARS/ml sample. Reproducibility of TBARS measurement in cell-free vitreous fluid samples had been previously verified to justify the single-value analysis which was necessary due to small available sample volumes. Additionally, the determined HNE-protein conjugates complement and support the TBARS data.

### 2.5. Measurement of Nitric Oxide (NO) Production

The concentration of nitrite, a stable product of nitric oxide (NO), was measured in vitreous humor with the Griess method, as described in [10]. The resulting concentration was expressed as nmol nitrite/ml sample.

### 2.6. Cu,Zn-Superoxide Dismutase (SOD) and Catalase Activity

As the levels of reactive oxygen species in vitreous and aqueous samples resulted to be poorly detectable in preliminary experiments, we measured the activities of SOD and catalase activities as representative enzymes for local antioxidative defense, as described in [8, 11], in 50 *μ*l aqueous and vitreous supernatants.

Briefly, SOD activity was measured by a specific SOD assay (Trevigen, Tema Ricerca Srl, Bologna, Italy) using the xanthine/xanthine oxidase/nitroblue tetrazolium (NBT) system to generate and quantitate superoxide ion. SOD prevents superoxide-mediated reduction of NBT and lowers the yield of blue NBT-diformazan. 50 *μ*l of sample was added to 200 *μ*l of reaction mix containing xanthine solution, xanthine oxidase, and NBT and immediately assayed for total SOD and specific Cu,Zn-SOD activity at 560 nm using a Lambda 3 spectrophotometer (Bio-Rad Laboratories, Hercules, California). To evaluate the contribution of Cu,Zn-SOD activity to the total activity, Mn-SOD and Fe-SOD were inactivated by adding 100 *μ*l of chloroform/ethanol (37.5/62.5, *v*/*v*) to the supernatant before assay. Cu,Zn-SOD activity was expressed as units per ml of supernatant.

Catalase activity was measured in 50 *μ*l aqueous and vitreous supernatants as reduction of H_2_O_2_ at 25°C by the spectrophotometric method [11] at 240 nm on UV/Vis spectrophotometer. The catalase-specific activity was expressed as units per ml of supernatant.

### 2.7. 4-Hydroxynonenal (HNE)-Protein Conjugate Assay

The accumulation of 4-HNE-protein conjugates represents the local oxidative status and lipoperoxidation process. At high concentration, 4-HNE is a bioactive toxic end-product of lipoperoxides that is reported as important pathomechanistic factor in many disease [12, 13]. As to its exceptional reactivity, its conjugates with proteins can be assessed by specific antibodies against the conjugate in western blot. Proteins were extracted from 30 *μ*l of vitreous and 30 *μ*l aqueous humor samples in presence of a protease inhibitor cocktail (Complete®, Roche Diagnostics S.p.A., Milano, Italy) in order to inhibit a broad spectrum of serine-, cysteine-, and metalloprotease activities, centrifugated at 2000 ×*g* at 4°C for 10 min to eliminate insoluble residues, and quantified for protein content by Bio-Rad Protein Assay (Bio-Rad, Hercules, CA, USA). As previously described [8], protein was extracted with SDS-containing modified Laemmli buffer (final concentrations of Tris-HCl 60 mM, EDTA 1 mM, 5% glycerol, and SDS 2%), pH 6.8, at 95°C for 5 min. Aliquots were kept at −20 C until use and *β*-mercaptoethanol (5% *v*/v, final concentration) was added before SDS-PAGE. The extracted proteins were requantified, loaded at 20 *μ*g/lane, and separated in a 10% polyacrylamide/acrylamide (*w*/*v*) SDS-PAGE. Separated proteins were transferred onto a nitrocellulose membrane (Amersham Biosciences, Fairfield, Connecticut), and equality of loaded and transferred protein amounts and of protein pattern of all sample lanes was verified by Ponceau S staining. Image acquisition, protein band intensity quantification, and comparison of lanes were performed using ImageJ software (version 1.46, Wayne Rasband, National Institutes of Health, Bethesda, MD, USA). Membranes were blocked with bovine serum albumin (BSA; dissolved at 5% (*w*/*v*) in phosphate-buffered saline containing 0.1% (*v*/v) Tween) for 1 h and subjected to the mouse monoclonal anti-4-HNE-conjugate antibody at 1 : 2000 dilution (clone HNEJ-2, Abcam, Cambridge, UK) overnight at 4 C [11, 14, 15]. Then membranes were washed with PBS-Tween 0.1% and incubated with the secondary anti-mouse horseradish peroxidase-conjugated antibody (Amersham, Bucks, United Kingdom) at 1 : 20000 dilution for 1 h at room temperature. The PBS-Tween 0.1%-washed membranes were analyzed for antibody-positive bands which were visualized by enhanced chemiluminescence (ECL) and acquired and quantified with Chemidoc MP equipment (Bio-Rad Laboratories, Hercules, California), using the PDQuest software (Bio-Rad, version 7.2) according to manufacturer's instructions. 4-HNE-modified human serum albumin (4-HNE-HSA) [14, 15] was prepared as reference sample for normalization of sample conjugate values. For this, 0.1 *μ*g 4-HNE-HSA was run in parallel with samples in each gel. For densitometry analysis, the values obtained for all 4-HNE-positive bands of a sample were referred to the reference 4-HNE-HSA band, summarized in each lane, and expressed as 4-HNE arbitrary units for each patient.

### 2.8. Hyaluronic Acid (HA) Assay

HA assay was performed in 20 *μ*l of vitreous samples using the human hyaluronic acid AlphaLISA® immunoassay kit according to the manufacturer's instructions (PerkinElmer, Waltham, Massachusetts, United States). The assay shows no cross-reactivity with human collagen Type I, Type II, Type III, and fibronectin. Absorbance was measured at 615 nm with the EnSight instrument (PerkinElmer, Waltham, Massachusetts, United States). Kaleido version 2.0.3058.126 and GraphPad Prism (5.01) software were used for data acquisition and elaboration. HA levels were expressed as ng HA/*μ*l of vitreous samples.

### 2.9. Statistical Analysis

Data were presented as mean ± standard error of the mean (SEM), and the results were checked for normal distribution and analyzed by one-way analysis of variance (ANOVA) followed by Tukey's test. Statistical significance level was set at *p*=0.05.

## 3. Results

**Figure 1:**
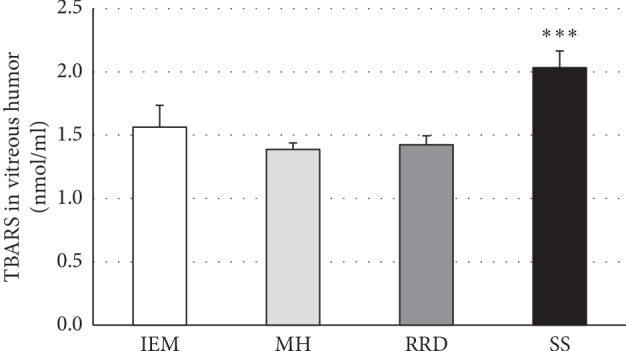
High thiobarbituric acid-reactive substances (TBARS) levels in vitreous humor of patients with synchysis scintillans as compared to other vitreoretinal diseases. Columns show means ± SEM of TBARS measured in vitreous humors of patients with idiopathic epimacular membrane (IEM; *n* = 12), macular hole (MH; *n* = 5), rhegmatogenous retinal detachment (RRD; *n* = 4), and synchysis scintillans (SS; *n* = 16). Significant difference between SS and other patient groups is indicated by *∗∗∗*, at *p* ≤ 0.001.

In human, vitreous TBARS levels are significantly higher by approximately 30% in patients affected by synchysis scintillans compared to patients who underwent *pars plana* vitrectomy for several vitreoretinal disorders without synchysis such as idiopathic epimacular membrane (IEM), macular hole (MH), or rhegmatogenous retinal detachment (RRD) (Figure 1), indicating an increased lipid peroxidation.

**Figure 2:**
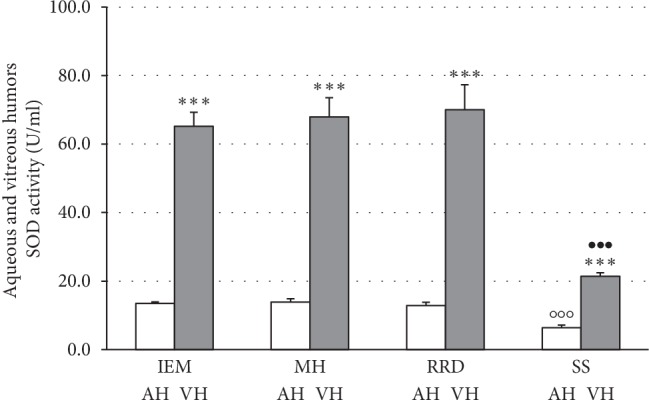
Low Cu,Zn-superoxide dismutase (SOD) activity in aqueous and vitreous humors of patients with synchysis scintillans as compared to other vitreoretinal diseases. Columns show means ± SEM of SOD activities measured in aqueous (AH) and vitreous humors (VH) of patients with vitreoretinal diseases without synchysis scintillans, i.e., idiopathic epimacular membrane (IEM; *n* = 12), macular hole (MH; *n* = 5), rhegmatogenous retinal detachment (RRD; *n*4), or patients with synchysis scintillans (SS; *n* = 16). Measurements were performed in duplicate. Significant differences are shown by *∗∗∗* at *p* < 0.001 between vitreous and aqueous humors, by ○○○ at *p* < 0.001 between SS and any of vitreoretinal patient groups in the aqueous humor, and by ••• at *p* < 0.0001 between SS and any of vitreoretinal patient groups in the vitreous humor.

**Figure 3:**
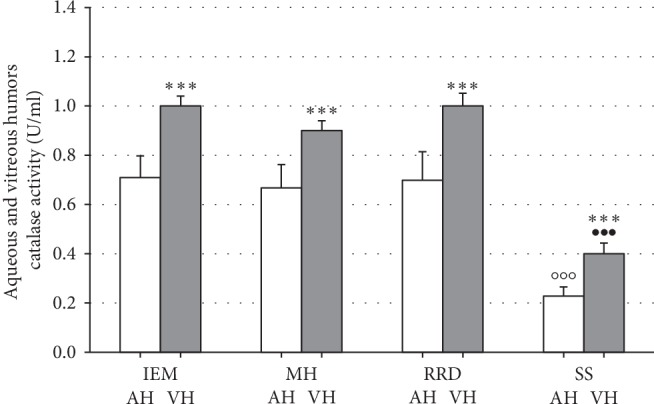
Low catalase activity in aqueous and vitreous humors of patients with synchysis scintillans as compared to other vitreoretinal diseases. Columns show means ± SEM of catalase activities measured in aqueous (AH) and vitreous humors (VH) of patients with vitreoretinal diseases without synchysis scintillans, i.e., idiopathic epimacular membrane (IEM; *n* = 12), macular hole (MH; *n* = 5), rhegmatogenous retinal detachment (RRD; *n*4), or patients with synchysis scintillans (SS; *n* = 16). Measurements were performed in duplicate. Significant differences are shown by *∗∗∗* at *p* < 0.001 between vitreous and aqueous humors, by ○○○ at *p* < 0.0001 between SS and any of vitreoretinal patient groups in the aqueous humor, and by ••• at *p* < 0.0001 between SS and any of vitreoretinal patient groups in the vitreous humor.

We observed a significant decrease in antioxidant activities of Cu,Zn-superoxide dismutase and catalase, in both aqueous and vitreous humor, in the synchysis scintillans group as compared to patients affected by any other vitreoretinal disorder under study (Figures 2 and 3). Comparing the different types of vitreoretinal diseases, there were no significant differences in the antioxidant enzyme activities (Figure 2 and 3) neither in the aqueous nor in the vitreous compartment, although SOD and catase activities are significantly highter in the vitreous compartment than in the aqueous one, being far more expressed in the vitreous compartment than in the aqueous one.

**Figure 4:**
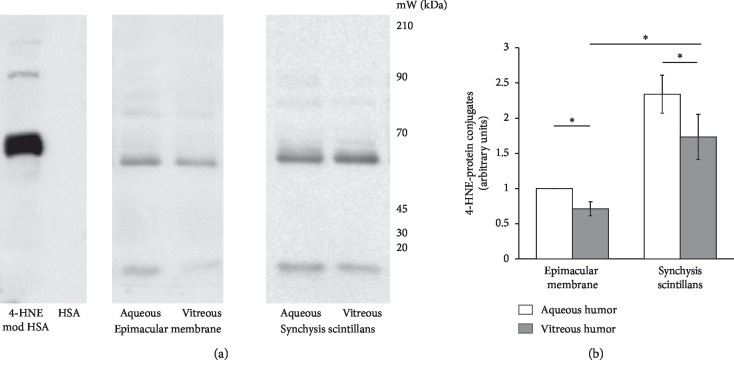
High 4-HNE-protein conjugates in aqueous and vitreous humors of patients with synchysis scintillans as compared to other vitreoretinal diseases. (a) Representative pattern of 4-HNE-protein conjugates extracted from the vitreous and aqueous humors of patients affected by idiopathic epimacular membrane or synchisis scintillans. Protein extracts were electrophoretically separated and western blotted, using antibody against anti-4-HNE-conjugates: purified human serum albumin (HSA) and 4-HNE-conjugated HSA (4-HNE-mod HSA) were used as negative and positive controls, respectively, to test the specificity of the method. The protein bands of the analyzed humors were quantified (see Section 2), and the values, expressed as arbitrary units, are represented in (b) as means ± SEM. Significant differences are shown by *∗* at *p* < 0.01.

As no differences in TBARS, SOD, and catalase activity levels were detectable between patient groups without synchysis scintillans, IEM patients were chosen to serve as representative controls for the synchysis scintillans group in 4-HNE-protein conjugate detection by western blotting. In accordance with the TBARS data and antioxidative enzyme activities, the level of proteins that were conjugated with the lipoperoxidation product 4-HNE (Figure 4) was significantly higher in aqueous versus vitreous humors of synchisis scintillans patients, whereas both the aqueous and vitreous supernatants derived from synchisis scintillans patients contained a significantly higher level of 4-HNE-protein conjugates than those from vitreoretinal patients.

**Figure 5:**
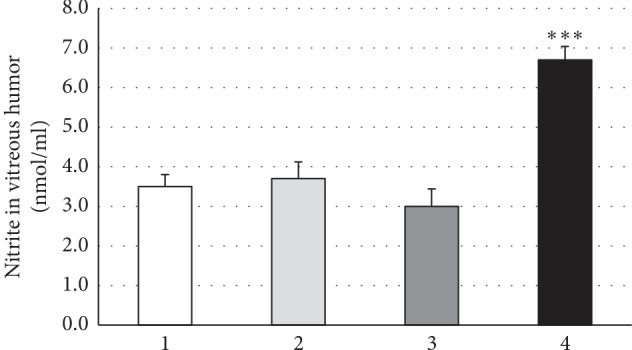
High nitrite levels in vitreous humors of patients with synchysis scintillans as compared to other vitreoretinal diseases. Columns show means ± SEM of nitrite concentrations in vitreous humors of patients with idiopathic epimacular membrane (IEM; *n* = 12), macular hole (MH; *n* = 5), rhegmatogenous retinal detachment (RRD; *n* = 4), or synchysis scintillans (SS; *n* = 16). Nitrite concentration was determined in duplicate per patient and only in vitreous humor due to small available sample volumes. Significant difference between SS and all other patient groups is indicated by *∗∗∗* at *p* ≤ 0.001.

**Figure 6:**
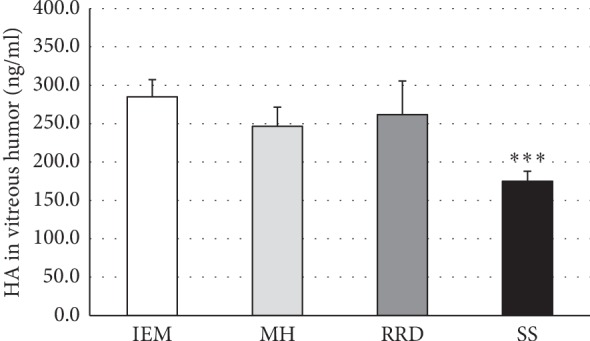
Low hyaluronic acid (HA) levels in vitreous humors of patients with synchysis scintillans as compared to vitreoretinal diseases without synchysis scintillans. Columns show means ± SEM of hyaluronic acid concentrations in vitreous humors of patients with idiopathic epimacular membrane (IEM; *n* = 12), macular hole (MH; *n* = 5), rhegmatogenous retinal detachment (RRD; *n* = 4), or synchysis scintillans (SS; *n* = 16). HA concentration was determined in duplicate per patient in vitreous humor. Significant difference between SS and all other patient groups is indicated by *∗∗∗* at *p* ≤ 0.001.

Moreover, the vitreous nitrite levels in synchisis scintillans patient group were about twice as high as in patients affected by various vitreoretinal disease (Figure 5), whereas the vitreous HA amount was significantly less in synchisis scintillans patients when compared to vitreoretinal disease patient groups (Figure 6).

## 4. Discussion

Although the vitreous accounts for about 80% of the globe's eye volume and plays a crucial role in the pathogenesis of various eye disorders, its physiologic role has been underestimated by ophthalmologists for long. The mechanism of vitreous liquefaction is not well understood, mainly due to the few studies either in patients or postmortem studies. Nevertheless, clinical evidence demonstrates that liquefaction in conjunction with weakening of the vitreous cortex-internal limiting laminar (ILM) adhesion, mostly when liquefaction occurs faster than the detachment of the vitreous cortex or when an abnormal adhesion of the vitreous cortex to the ILM occurs, results in PVD [16]. Previous reports indicated that intravitreal oxidative stress provokes disruption of hyaluronic acid leading to vitreous liquefaction and ultimately PVD [17]. Consequently, PVD leads to the abnormal vitreoretinal interface and vitreomacular traction [18]. Particularly, on the basis of the direction of traction, tractional force centripetal (inward toward the fovea) causes macular pucker, whereas tangential traction in a centrifugal direction (outward from the fovea) determines a macular hole [16].

Also, IEM with largely collagen structures caused by glial cells, hyalocytes, and myofibroblasts proliferation in the macular area usually results in PVD process [19–21], through a defect in the ILM and accompanied by a decrease in visual acuity and disturbing metamorphopsia, photophobia, and micropsia [22]. Furthermore, the rhegmatogenous retinal detachment (RRD) is the most common type of detachment, and it is characterized by a full-thickness break in the neural retina leading to vitreous fluid influx into the subretinal space. Thus, RRD causes photoreceptor cells to lose direct contact with the retinal pigment epithelium (RPE), exposes them to liquid vitreous humor, and forms a diffusional barrier to the flux of oxygen and nutrients coming from the RPE and the choroid vasculature. The resultant hypoxia and nutrient deprivation are evidenced by the upregulation of hypoxia-inducible factors (HIFs) in experimental models of RRD [23]. Moreover, recently, Kiang et al. have documented that elevated levels of cytokines and growth factors in the vitreous of retinal detachment patients contributed to the onset of proliferative vitreoretinopathy in patients with retinal detachment [24].

Next to these vitreoretinal diseases, the synchysis scintillans is a degenerative condition of the eye resulting in liquefied vitreous humor, and it is formed of multiple vitreous opacities that are flat, mobile, and golden brown in color with the collagen displaced peripherally [16, 25, 26]. Synchysis scintillans is not well studied from a biomolecular point of view as it is a rare condition, also known as “cholesterolosis bulbi,” as the presence of refringent cholesterol crystals was demonstrated in these opacities [16]. The developed cholesterol crystals, clinically detectable anterior chamber, is to be attributed to different pathogenetic mechanisms following the breakdown of vitreous and anterior chamber haemorrhage or deriving from the subretinal fluid of a chronic total retinal detachment in the absence of any intraocular haemorrhage resulting from phacolysis and associated with a marked neutrophil response [27].

In this paper, we propose OS and lipoperoxidation as causative factors in cholesterol crystal formation. It is well known that oxidative stress is a common denominator link for the major pathways which are involved in the disease development and progression for clinical complications. Indeed, there is increasing evidence indicating that persistent OS contributes to the development of many ocular diseases, among which are noncancer ocular diseases (dry eye syndrome, corneal and conjunctive diseases, cataract, glaucoma, retinitis pigmentosa, diabetic retinopathy, and autoimmune and inflammatory uveitis) and cancer ocular diseases (melanoma, retinoblastoma, and lymphoma) [4]. Moreover, the increase in vitreous cytokine mRNA expression and concentrations in human RRD shows that oxidative stress status is a significant component of the retina's response in photoreceptor degeneration [28]. The eye is one of the major targets of the ROS/RNS attack due to exposition on several environmental factors like light exposure, ultraviolet rays, ionizing radiation, chemical pollutants, irritant, high pressure of oxygen, pathogenic microbes, and also Q-switched Nd:YAG treatment [8], which are able to shift the redox status of a cell toward oxidizing conditions [29]. The role of OS in synchysis scintillant condition in not studied well even if the failure in the efficiency of antioxidant system as one of the causes of vitreous opacities was supposed as early as in 1929 [30]. Notably, ROS and lipoperoxidation products including HNE were shown to be causative for cholesterol monohydrate crystal formation in supersaturated model bile [31]. Moreover, the lipid peroxidation is known to promote formation of crystalline structures of free cholesterol [32], suggesting the high level of HNE in synchysis patients could be mechanistic for cholesterol crystal formation. Additional indirect evidence of the role of lipoperoxidation in synchysis scintillans pathogenesis is that rabbits fed a diet rich in cholesterol developed synchysis scintillans with abnormally high deposition of extravascular lipid material [33] which is a good substract for lipoperoxidation.

In the present study, we highlighted that, in all the investigated eye pathologies, there is an oxidative status condition measured as presence of TBARS levels and 4-HNE products, in both aqueous and vitreous humors, that is, significantly more evident in patients affected by vitreous disease compared to those having vitreoretinal diseases. Moreover, in synchisis scintillans patients, the vitreous nitrite levels were significantly higher compared to the nitrite levels in patients affected by various vitreoretinal diseases. Extensive research during the past three decades has demonstrated the mechanisms by which an imbalance in the redox status of prooxidant/antioxidant reactions in cells with advantage of prooxidant reactions can cause peroxidation of nucleic acids, bases, lipids, proteins, and carbohydrates, thus resulting in their damage leading to chronic inflammation and causing tissue dysfunction [29]. Our data show that the antioxidative activities of SOD and catalase are significantly lower in synchisis scintillans group compared with patients affected by vitreoretinal disorders, confirming that dysregulation of SOD and catalase activities at the vitreous base may be critical in the pathophysiology of oxidative stress-related vitreoretinopathies. Moreover, our results demonstrated that, in synchisis scintillans patients, the amount of HA was significantly less compared to patients affected by vitreoretinal disease. Free radicals generated by metabolic reactions as well as the presence of cholesterol crystals could alter hyaluronan and/or collagen structure and trigger a dissociation of collagen and hyaluronan molecules [34], which ultimately results in vitreous liquefaction [35, 36].

In our opinion, it is plausible that the development of synchisis scintillans could be attributed to frequent or chronic proinflammatory states, as allergic infections never diagnosed within mild symptoms, that could explain an accumulation of activated macrophages-derived cholesterol-forming crystals in the eye [37]. As a consequence, even if synchisis scintillans is sometimes considered a clinically insignificant condition, the retina is certainly exposed to irregular light exposure that could result in retinal damage and degenerations [38]. Moreover, also the cholesterol crystals could trigger inflammasome-mediated innate responses activation leading to retinal damage [36, 39] and adding additional clinical significance to synchisis scintillans.

A better understanding of response to oxidative stress related to eye pathologies will lead to new therapeutic approaches for the prevention or amelioration of age-associated degenerative diseases.

## Data Availability

The data used to support the findings of this study are available from the corresponding author upon request.
